# Forgone and delayed care in Germany– inequalities and perceived health risk of unmet need

**DOI:** 10.1186/s12939-025-02483-6

**Published:** 2025-05-06

**Authors:** Jens Klein, Daniel Lüdecke, Olaf von dem Knesebeck

**Affiliations:** Institute of Medical Sociology, University Medical Center Hamburg-Eppendorf, Martinistr. 52, 20246 Hamburg, Germany

**Keywords:** Unmet need, Forgone care, Delayed care, Social inequalities, Access, Equity in health care, Multilevel analyses

## Abstract

**Background:**

Subjective unmet need is an established indicator of unequal access to medical care and is often measured by delaying and forgoing medically necessary treatment. Research on delayed and forgone care among the general population in Germany including different reasons, social deprivation measures, and the perceived health risk of unmet need is sparse. This study aims to examine reasons, inequalities, and health-related consequences of unmet need in terms of delayed and forgone care.

**Methods:**

A cross-sectional online survey was carried out based on a randomly drawn sample of the German adult population in December 2022 (*N* = 2,201). Respondents were asked whether medical treatments were delayed or forgone in the past 12 months due to different reasons (waiting time, travel distance, financial costs). If unmet need was indicated, the respondents were subsequently asked about their perception of related health risks. Associations with individual social (sex, age, migration history, education, income) and regional factors (social deprivation) as well as insurance status were examined using multilevel logistic regressions analyses.

**Results:**

Among *N* = 1,955 respondents who indicated need for medical care, 30% reported at least one reason for forgone care (waiting time 23%, financial costs 11%, travel distance 9%). In terms of delayed care, highest rate was found for waiting time (34%). Multilevel analyses revealed significant associations of unmet need with female sex, younger age, lower education, lower income, and statutory health insurance. Associations varied depending on the reason for unmet need. Differences in regional social deprivation were particularly found for forgone care due to distance. Between half and nearly two-thirds of the participants reported worsening of symptoms in case of unmet need. Associations with social characteristics were inconsistent.

**Discussion:**

Unmet need is a prevailing issue in Germany and associated with perceived worsening of health, various indicators of social inequality, and health insurance. Reducing waiting times (e.g. through the further development of appointment service centres) and private co-payments as well as ensuring health care provision in deprived areas can contribute to a decrease of barrier-related unmet need and health risks. However, more in-depth studies are required to account for the complex nature of health care access.

## Introduction

Unmet need is defined as “differences between services judged necessary to deal appropriately with health problems and services actually received” [1]. In case of subjective unmet need, a person perceives need of health care but does not receive or use respective services due to access barriers beyond his or her control [2]. This study was based on the concept of subjective, not-chosen unmet need which is an established measure of health care access and its underlying barriers [2–4]. Different reasons for this kind of unmet need are distinguished. Most relevant from a health policy perspective and predominantly included in previous research are reasons referring to availability (waiting time, travel distance) and affordability (financial costs) which are also central components in the conceptual framework of health care access by Levesque et al. (2013) [2–7]. Apart from these main reasons, further individual rationales for unmet need are work and family commitments, fear of doctor and treatment, preferring to wait and see, and not knowing any good doctor [8]. To ascertain unmet need, measures of forgone and delayed care have been used in various surveys [5, 9–12].

Prevalence of unmet need varies across different countries. According to EHIS (European Health Interview Survey) data among 30 OECD and EU countries, on average, 28% of adults reported unmet need in a period of 12 months due to financial costs (16%), long waiting times (18%), or travel distance/transport problems (4%) [5]. Moreover, all types of unmet need were much more pronounced among the least wealthy in nearly all countries under study [5]. Even though levels of unmet need vary across surveys due to different methods and approaches [6, 12, 13], other international surveys showed similar patterns [4, 6, 14–16]. Apart from income, further social predictors are relevant for unmet need. Women, younger persons, people with limited insurance coverage, lower occupational position, and migration history were more likely to report forgone care [4, 14, 15, 17–19]. Educational inequalities were less pronounced [14, 15]. Although unmet need is an established measure in health services research [3, 5], empirical studies on negative health effects of subjective unmet need are less common. Two longitudinal studies from the U.S. showed associations between delayed or forgone care and adverse health outcomes [20, 21]. Similar associations were found in Europe and Asia [22–24]. Current data regarding delayed and forgone care due to the COVID-19 pandemic in the Netherlands reported negative health effects as a result of postponed care [25, 26].

### The case of Germany

The German health care system is based on a social health insurance system and is primarily funded by insurance contributions. Health insurance is compulsory and characterized by a dual structure of statutory health insurance (SHI) and substitutive private health insurance (PHI). Citizens with an income over a certain limit, self-employed, and public servants can choose a PHI for substitutive full coverage. Around 11% of the population is covered through PHI. Privately insured people experience some benefits compared to those covered by SHI (e.g. shorter waiting times) [27]. German studies of unmet need differ in terms of populations under study and survey methods. Overall, 32% reported forgone or delayed care due to waiting time (25%), travel distance (4%), or financial costs (14%), with higher rates among lower income groups (EHIS wave 2 data). Income-related inequalities for unmet need were particularly high for Germany [5]. Further surveys including data for Germany (e.g. EU-SILC, SHARE) varied in the level of unmet need, but also showed various social inequalities [9, 28–32]. Data about differences due to insurance status are rare. Finally, health risks of subjective unmet need in Germany are largely unknown.

Against this background, the present study includes following additional contributions: First, current detailed data is provided. Unmet need among the German population is a prevailing issue due to current changes and shortages in health services (e.g. supply of outpatient care in rural or deprived areas) [33–36]. The present study provides first detailed analyses after the COVID-19 pandemic which is known to have a great impact on delayed and forgone health care utilization in various European countries and the U.S [37, 38]. Second, social regional deprivation was included as it was shown that residing in more deprived urban or rural districts was associated with lower general practitioner (GP)availability in Germany [34, 35]. Accordingly, comprehensive multilevel analyses were conducted. Third, delayed and forgone care were separately analysed for an improved assessment of barrier-related unmet need in the German population. Fourth, associations between health insurance and different reasons for unmet need were included. Fifth, self-perceived health risks due to unmet need among the general population were additionally ascertained. Thus, the study aimed to examine the magnitude of unmet need, its reasons, and health-related consequences. In multilevel analyses, individual and regional social determinants of unmet need and perceived health risk were additionally examined. Accordingly, the following research questions were addressed:

1. To what extent are medical treatments delayed and forgone in the German general population due to different reasons (waiting time, travel distance, financial costs)?
2. Are there social inequalities in delayed and forgone care, and is regional deprivation associated with delayed and forgone care?
3. How does the population perceive health risks after delayed or forgone care?
4. Are there social inequalities in these perceptions?

## Methods

### Study design and population

The questionnaire was initiated by the research team. Analyses were based on a cross-sectional online survey that was conducted by a social research institute (forsa) in winter 2022/23. An adult population sample (age ≥ 18 years) was randomly drawn from a panel. This panel comprises a sample of the population living in Germany which was recruited via telephone using a dual-frame approach that included landline as well as mobile phone numbers. It is regularly refreshed and currently consists of about 150,000 people. 5,619 German-speaking individuals were randomly selected from the panel and invited to participate in the present survey via email. After three reminders, *N* = 2,201 individuals participated. Based on power calculations (statistical power = 0.8; α = 0.05; Cohen’s d = 0.2), a sample size of about *N* = 2,200 was aimed at to identify statistically reliable differences between various subgroups under study (e.g. privately and statutorily insured). The sample was weighted for age, sex, federal state, and education (using the iterative proportional fitting approach [39]) according to the official statistics provided by the Federal Statistical Office of Germany [40]. Thus, the weighted sample adequately represents the adult population in Germany regarding these sociodemographic characteristics. All procedures were in accordance with the ethical standards of the institutional and/or national research committee and with the 1964 Helsinki Declaration and its later amendments or comparable ethical standards. The survey was approved by the Local Psychological Ethics Committee at the Center for Psychosocial Medicine, University Medical Center Hamburg (No. LPEK-0563).

### Outcome variables

Unmet need was surveyed as follows: Initially, the participants were asked if there had been a need for examination or treatment. If not, participants were excluded from the analyses. Subsequently, two questions were asked regarding delayed care [41, 42]: (1) “Have you experienced delay in getting health care in the past 12 months because the time needed to obtain an appointment was too long?”, and (2) “Have you experienced delay in getting health care in the past 12 months due to distance or transportation problems?”. Respective response options were “yes”, “no”, “don’t know”/”not specified”. In terms of forgone care, three questions were asked including the most relevant, system-related reasons [43]: “During the past 12 months did it ever happen that you did not get the medical treatment you needed because you could not pay for it?”, (2) “During the past 12 months did it ever happen that you did not get the medical treatment you needed because the treatment you needed was not available where you live or nearby?”, and (3) “During the past 12 months did it ever happen that you did not get the medical treatment you needed because the waiting time/waiting list was too long?”. Again, response options were “yes”, “no”, “don’t know”/”not specified”. Responses of these three types of foregone care were combined to assess if at least one type of forgone care was experienced in the past 12 months. In case of reporting delayed or forgone care, respondents were requested to assess related health risks: “If so, has this made your symptoms worse?” (response options: “yes”, “no”, “don’t know”/”not specified”). To consider all three reasons of system-related unmet need, this question was asked in terms of delay due to waiting time, delay due to distance, and forgoing treatment due to costs.

### Independent variables

The following individual social characteristics of the respondents were considered as predictors: age, sex, education, income, and migration history. Age was categorized into three age groups: 18–40 years, 41–59 years, and ≥ 60 years. Educational level was assessed according to the established CASMIN educational classification which is a hierarchically structured measurement of certificates including the general and vocational qualifications [44]. The nine original CASMIN-levels were merged into three educational groups: low (levels 1a, 1b and 1c), intermediate (2a and 2b), and high (2c_gen, 2c_voc, 3a and 3b). Monthly net household income was equalized to consider household size and composition and further divided into tertiles. In terms of migration history, respondents were classified into three groups: people who immigrated themselves (1st generation migrants); people who were born in Germany, but whose parents (one or both) had immigrated (2nd generation migrants), and those without a migration history. Furthermore, insurance status (statutory/private) was introduced.

As regional disparities were also shown to be an important determinant of health care access [33–35, 45] the German Index of Social Deprivation (GISD) was introduced on the area level [46]. This index uses administrative data of education (e.g. proportion of employees with university degree and without qualification), employment (e.g. unemployment rate, gross wage and salary), and income (net household income, debtor quota, tax revenue) at the district and municipality level. In the present analyses, classification was based on postal codes.

### Analyses

As *n* = 246 (11.2%) of the participants indicated that there was no need for examination or treatment in the past 12 months, a remaining sample of *N* = 1,955 was included in the analyses. First, prevalence of unmet need (i.e. delayed and forgone care due to different reasons) and perceived health risk due to unmet need was calculated. Second, multilevel logistic regressions were carried out to consider potential predictors of unmet need on individual and area level. In fully adjusted models, associations between delayed and forgone care and all individual social characteristics (sex, age, migration history, education, income) and insurance status were calculated. The GISD was used as level 2 unit in the mixed model (random intercept) to account for differences in regional deprivation. Odds ratios (OR), 95%-confidence intervals (95%-CI), and p-values are documented. Variance, intraclass correlation coefficient (ICC), and the median odds ratio (MOR) are also reported to provide information about the contribution of the area level to the explained variance of the model. For a more intuitive interpretation of the area level variance, the MOR was introduced by Merlo et al. [47] and was calculated as a measure of the mean variation in unmet need between the different deprived groups [48]. The same procedures of multivariate analyses were conducted for perceived health risk due to delayed and forgone care as dependent variables. Due to a small number of cases, delay due to waiting time and delay due to distance were matched for these analyses. Analyses were carried out using the Statistical Package for the Social Sciences SPSS 29 [49].

## Results

Detailed information about the sample characteristics are shown in Table 1.

**Table 1:** Sample characteristics (*N* = 1,955^1^)

		*n* (%)
Sex *(0)*	Female	1,009 (51.6)
	Male	946 (48.4)
Age groups *(0)*	18–40 years	614 (31.4)
	41–59 years	654 (33.5)
	≥ 60 years	687 (35.1)
Migration history *(34)*	No	1,479 (77.0)
	1st generation	147 (7.6)
	2nd generation	295 (15.3)
Education^2^*(60)*	High	709 (37.4)
	Intermediate	595 (31.4)
	Low	591 (31.2)
Income^3^*(288)*	upper tertile (≥ 2250€)	558 (33.4)
	middle tertile	560 (33.6)
	lower tertile (≤ 1625€)	550 (33.0)
Social deprivation^4^*(5)*	1st quintile (least deprived)	430 (22.1)
	2nd– 4th quintile	1,125 (57.7)
	5th quintile (most deprived)	394 (20.2)
Health insurance *(3)*	statutory	1,698 (87.7)
	private	239 (12.3)

^1^Weighted data; number of missing cases in italics

^2^Educational classification according to CASMIN

^3^Monthly net household income (equalized)

^4^German Index of Social Deprivation (GISD)

Table 2 shows magnitudes of unmet need and perceived health risk due to unmet need within the past 12 months. Delayed (34.2%) and forgone care (23.1%) due to waiting time were most frequently mentioned. About 10% of the sample indicated forgone care due to distance and financial costs and about 6% reported delayed care due to distance. Finally, approximately 31% experienced at least one of the three types of forgone care in the past 12 months. If unmet need was indicated by the respondents, more than a half up to two-thirds reported a worsening of their own health as a consequence.

**Table 2:** Unmet need and perceived health risk due to unmet need within the past 12 months (*n* = 1,941^1^): N (%)

Delayed care		*N* (%)
Waiting time	No delay	1,277 (65.8)
	Delay	663 (34.2)
	*Thereof * ***Perceived health risk***	*356 (53.7)*
Distance	No delay	1,821 (93.8)
	Delay	120 (6.2)
	*Thereof * ***Perceived health risk***	*78 (65.0)*
**Forgone care**		
Waiting time	No forgoing	1,486 (76.9)
	Forgoing	446 (23.1)
Distance	No forgoing	1,753 (90.7)
	Forgoing	180 (9.3)
Financial costs	No forgoing	1,727 (89.2)
	Forgoing	209 (10.8)
	*Thereof * ***Perceived health risk***	*132 (63.2)*
At least one type of forgone care	No forgoing	1,340 (69.5)
	Forgoing	588 (30.5)

^1^Some case numbers do not sum up to 1,941 due to missing cases

Table 3 shows the results of multilevel logistic regressions for delayed care due to waiting times and distance as dependent variables. All predictors and covariates were introduced simultaneously. Female sex (OR: 1.49 and 1.75), younger age (OR: 1.46 to 2.37), a statutory insurance (OR: 3.51 in case of waiting time), and low income (OR: 1.68 in case of distance) played a statistically significant role for reporting delayed care, while migration history and education were not significantly associated with delayed care. Social deprivation on the area level did not show notable variations.

**Table 3:** Delayed care within the past 12 months: multilevel logistic regression analysis^1^

	Delay due to…			
	waiting time		distance	
***Individual level***	OR (95%-CI)	p	OR (95%-CI)	p
Sex (ref: male)				
female	**1.75 (1.42–2.15)**	**< 0.001**	**1.49 (1.02–2.18)**	**0.040**
Age (ref: ≥60 years)				
18–40	**2.26 (1.70–3.01)**	**< 0.001**	**2.37 (1.39–4.06)**	**0.002**
41–59	**1.46 (1.13–1.90)**	**0.004**	1.44 (0.86–2.41)	0.162
Migration history (ref: no)				
1st generation	1.08 (0.72–1.62)	0.697	0.70 (0.30–1.63)	0.407
2nd generation	0.94 (0.71–1.25)	0.666	0.82 (0.49–1.40)	0.468
Education (ref: high)				
intermediate	0.92 (0.71–1.20)	0.534	1.11 (0.69–1.78)	0.661
low	1.01 (0.76–1.35)	0.930	1.36 (0.80–2.30)	0.258
Income (ref: upper tertile)				
2nd	1.03 (0.80–1.33)	0.836	1.01 (0.60–1.68)	0.986
3rd	0.94 (0.72–1.21)	0.617	**1.68 (1.05–2.68)**	**0.030**
Health insurance (ref: private)				
statutory	**3.51 (2.33–5.30)**	**< 0.001**	1.50 (0.72–3.08)	0.273
***Area level***				
Social deprivation^2^				
Variance	0.018		0.000	
ICC^3^	0.004		0.000	
MOR^4^	1.14		1.00	
Observations	1,708		1,708	

^1^All variables in the models are adjusted for each other

^2^German Index of Social Deprivation (GISD)

^3^Intraclass correlation coefficient

^4^Median odds ratio

Significant associations (*p* < 0.05) are bold

Multilevel regression analyses of the reasons for forgone care are shown in Table 4. Young age was associated with reporting forgone care for any of the mentioned reasons. Female sex, lower income, and lower education were significantly associated with forgone care due to financial costs (OR: 1.54 to 2.57). Insurance status was particularly related to forgone care due to waiting time and distance (OR: 2.20 and 2.39). Indicating at least one of the three types of forgone care was significantly associated with being female (OR: 1.59), younger (OR: 1.60 to 2.49), less affluent (OR: 1.34 to 1.50), and statutorily insured (OR: 2.02). Social deprivation on the area level was related to forgone care due to distance (ICC: 0.018 and.

**Table 4:** Forgone care within the past 12 months: multilevel logistic regression analysis^1^

	Forgone care due to…						At least one type of forgone care	
	waiting time		distance		financial costs			
***Individual level***	OR (95%-CI)	p	OR (95%-CI)	p	OR (95%-CI)	p	OR (95%-CI)	p
Sex (ref: male)								
female	**1.64 (1.30–2.07)**	**< 0.001**	1.39 (0.99–1.96)	0.057	**2.05 (1.50–2.81)**	**< 0.001**	**1.59 (1.28–1.97)**	**< 0.001**
Age (ref: ≥60 years)								
18–40	**2.95 (2.12–4.12)**	**< 0.001**	**2.55 (1.58–4.10)**	**< 0.001**	**1.68 (1.10–2.56)**	**0.017**	**2.49 (1.84–3.36)**	**< 0.001**
41–59	**1.88 (1.38–2.56)**	**< 0.001**	1.55 (0.99–2.43)	0.058	1.42 (0.97–2.09)	0.071	**1.60 (1.22–2.11)**	**< 0.001**
Migration history (ref: no)								
1st generation	1.25 (0.80–1.94)	0.334	0.88 (0.43–1.80)	0.721	1.03 (0.55–1.92)	0.925	1.24 (0.82–1.88)	0.313
2nd generation	1.14 (0.84–1.55)	0.409	0.91 (0.57–1.47)	0.711	1.44 (0.97–2.13)	0.070	1.10 (0.82–1.48)	0.521
Education (ref: high)								
intermediate	0.95 (0.72–1.27)	0.746	**1.55 (1.02–2.36)**	**0.041**	**1.54 (1.04–2.28)**	**0.033**	1.16 (0.89–1.52)	0.276
low	1.24 (0.89–1.72)	0.198	1.42 (0.87–2.34)	0.164	**1.92 (1.25–2.96)**	**0.003**	1.35 (1.00-1.83)	0.053
Income (ref: upper tertile)								
middle tertile	1.18 (0.88–1.57)	0.270	1.37 (0.88–2.15)	0.168	**1.81 (1.19–2.78)**	**0.006**	**1.34 (1.02–1.76)**	**0.033**
lower tertile	1.08 (0.81–1.44)	0.613	1.51 (0.97–2.35)	0.071	**2.57 (1.70–3.88)**	**< 0.001**	**1.50 (1.15–1.97)**	**0.003**
Health insurance (ref: private)								
statutory	**2.20 (1.40–3.44)**	**0.001**	**2.39 (1.08–5.27)**	**0.031**	1.12 (0.64–1.98)	0.690	**2.02 (1.36–3.02)**	**< 0.001**
***Area level***								
Social deprivation^2^								
Variance	0.000		0.221		0.000		0.011	
ICC^3^	0.000		0.018		0.000		0.002	
MOR^4^	1.00		1.56		1.00		1.11	
Observations	1,683		1,683		1,683		1,683	

^1^All variables in the models are adjusted for each other ^2^German Index of Social Deprivation (GISD)

^3^Intraclass correlation coefficient ^4^Median odds ratio; significant associations (*p* < 0.05) are bold

Finally, Table 5 shows the results of the multilevel logistic regression analyses with perceived health risk due to delay and forgoing as dependent variable. Participants with lower age and with an own migration history perceived a greater health risk due to delay (waiting time/distance). In terms of perceived health consequences due to forgone care (financial costs), only medium age showed a significant association. Odds ratios suggested clear trends, even though p-values did not indicate significance.

**Table 5:** Perceived health risk due to delayed and forgone care: multilevel logistic regression analysis^1^

	Perceived health risk due to delayed care		Perceived health risk due to forgone care	
	(waiting time/distance)		(financial costs)	
***Individual level***	OR (95%-CI)	p	OR (95%-CI)	p
Sex (ref: male)				
female	1.17 (0.84–1.63)	0.359	1.30 (0.61–2.64)	0.523
Age (ref: ≥60 years)				
18–40	**1.83 (1.15–2.87)**	**0.010**	1.61 (0.65–3.97)	0.304
41–59	**1.84 (1.20–2.83)**	**0.005**	**2.91 (1.16–7.33)**	**0.024**
Migration history (ref: no)				
1st generation	**2.14 (1.08–4.23)**	**0.030**	0.65 (0.17–2.54)	0.536
2nd generation	1.02 (0.65–1.60)	0.926	0.61 (0.26–1.43)	0.253
Education (ref: high)				
intermediate	1.18 (0.78–1.77)	0.436	0.46 (0.18–1.19)	0.109
low	1.53 (0.95–2.46)	0.082	1.08 (0.39-3.00)	0.879
Income (ref: upper tertile)				
2nd	1.28 (0.85–1.94)	0.234	1.78 (0.62–5.11)	0.283
3rd	1.12 (0.74–1.69)	0.585	1.80 (0.65–4.97)	0.253
Insurance (ref: private)				
statutory	1.03 (0.47–2.23)	0.943	3.83 (0.96–15.25)	0.056
***Area level***				
Social deprivation^2^				
Variance	0.000		0.000	
ICC	0.000		0.000	
MOR	1.00		1.00	
Observations	613		183	

^1^All variables in the models are adjusted for each other

^2^German index of social deprivation (GISD)

^3^Intraclass correlation coefficient

^4^Median odds ratio

Significant associations (*p* < 0.05) are bold

## Discussion

In the present study, magnitude, reasons, inequalities, and self-perceived health risks of unmet medical care need (delayed and forgone care) were examined among the general population in Germany. Delayed care was reported by 34% (waiting time), and 6% (distance) respectively. Prevalence of forgone care varied depending on the three different reasons (waiting time 23%; financial costs 11%; distance 9%). At least one of the three types of forgone care was indicated by 31% of the respondents. Significant social predictors for delayed care were female sex, younger age, as well as statutory insurance. Forgone care was associated with female sex, age < 60 years, lower income, lower education, and statutory insurance. Some differences were shown depending on the particular reason for forgone care. More than a half up to nearly two-thirds reported a perceived health risk due to delayed and forgone medical treatments. The perception of health risk was less pronounced among older participants. Area level social deprivation was shown to be particularly relevant for forgoing medical treatment due to distance.

Differences in methods and approaches used to assess unmet need among different surveys hamper comparisons with previous research. More precisely, these differences refer to the population considered, the range of health services covered, the reasons for unmet needs, the wording of questions, and the inclusion of delayed and forgone care (as opposed to forgone care only) in the definition of unmet need [6, 12, 13]. Comparisons across different countries have to be drawn carefully as country-specific aspects of the organisation of health care provision and the way in which vulnerable groups are protected from charges have to be taken into account [4]. In terms of Germany, patterns in the present study are in line with former research, even though prevalences of forgone medical care were higher in our study [5, 14, 28]. For instance, the EU-SILC survey considered the total population surveyed and not exclusively people with health care need. Furthermore, the questions of the EU-SILC survey in Germany referred to unmet needs for *severe* illnesses which also results in under-estimation of magnitudes and inequalities [6, 13]. Overall, clear social inequalities in unmet need and its predictors (sex, age, income, but less education) of former research from Germany was confirmed by the present study, and additionally, inequalities regarding health insurance were shown [4, 5, 28]. Similar to previous results from Germany, a main reason for unmet need was waiting time. This is still a highly discussed issue in Germany as patients with a SHI have to wait longer for an appointment than privately insured patients [43, 44]. Indeed, associations between migrant history and unmet need were hardly found which could be due to different sample characteristics.

The magnitudes, inequalities, and perceived health risks of unmet need suggest important health policy issues and call for action. The findings showed that economic factors such as affluence, place of residence, and insurance coverage are important drivers of observed disparities in our study. Furthermore, women and younger persons constantly reported higher rates of unmet need. The results vary depending on the reason for unmet need. In terms of delayed and forgone care due to waiting time, statutorily insured patients clearly reported higher unmet need. Previous research showed significantly longer waiting times for an appointment in the German outpatient setting for patients with statutorily health insurance irrespective of social status [50–53]. In this context, it has to be kept in mind that doctors are allowed to charge higher fees for privately insured patients, which potentially creates incentives for preferred treatment. Thus, abolishing the coexistence of SHI and PHI is a highly discussed topic which is supported by the majority of the population [54], and would promote more health care equity in terms of waiting time [55]. A study among statutorily insured people hardly found associations between forgone care and social status indicators, but particularly with perceived discrimination related to health care (e.g. waiting times) [32]. To reduce this discrimination of statutorily insured regarding waiting times, appointment service centres for medical appointments were introduced in 2019. First evaluations regarding specialist care revealed relatively low use, but the ability to make urgent appointments, with average waiting times significantly lower than the legally set maximum waiting period [56]. Improved education about different possibilities in the health care system to seek for timely treatment could increase the use of such services.

Forgone care due to distance was also associated with statutorily health insurance, and additionally with regional social deprivation in our study. Regarding the latter, it was shown that residing in more deprived urban or rural districts was associated with lower GP availability [34, 35]. Moreover, a study has shown that a higher proportion of privately insured people in a region was associated with higher GP and specialist density [38]. This suggests an unequal distribution of outpatient care to the disadvantage of deprived areas and statutorily insured patients which is an important issue in health policy research [33, 57, 58]. Different incentives and strategies (e.g. facilitating job opportunities for third-country physicians, improving promotion in medical faculties, raising consciousness in students for rural primary care when applying for university) were introduced to increase the number of physicians in these areas [59–61]. However, evidence about the effectiveness of such strategies is poor.

Forgoing medical care due to financial costs was not related to insurance status, but particularly to income. In Germany, financial and material circumstances are associated with access to and utilization of care, and thus, result in increased unmet need [31]. Financial burden due to out-of-pocket payments were much more pronounced among less affluent patients and to a lesser extent among SHI patients which facilitates delay and forgone care of necessary medical treatments [31, 62]. Generally, SHI covers a wide range of benefits that are the same for all those insured. The share of private out-of-pocket funding is moderate, even though co-payments are required (e.g. for prescribed or over-the-counter drugs and therapies). Moreover, a better education about costs and possible refunds could be helpful. Physicians’ representatives also worry about increased unmet needs and highlighted their requests including better financing, streamlining of administration, and faster implementations of reforms [63]. Moreover, an overall consistent association with female sex and young age may indicate that these sub-groups have a stronger awareness of access barriers. The findings provide information about the perception of health-related consequences of unmet need. High proportions of people who reported a worsening of their health due to delay or forgoing show the importance to take notice of delayed and forgone care in further health care system development. Finally, when putting postponed care in relation to health care costs, additional benefits were found when diminishing unmet need. Associations were found between forgone and delayed medical care and significantly higher health care expenditures among a heart failure population in the U. S [64].

### Limitations of the study

There are some limitations of this study that have to be discussed. The sample was randomly drawn from a panel which was recruited offline. However, analyses were based on an online survey and only internet users and people with internet connection could be included. Furthermore, a selection bias cannot be ruled out as only about 39% of the invited persons participated. The distribution of social characteristics in our study compared to the general population was satisfying. Nevertheless, data was weighted by age, sex, federal state, and education according to the official statistics [40] using an iterative proportional fitting approach [39] to account for a potential bias. Moreover, analyses were restricted to individuals who were able to read German. This has to be especially kept in mind when evaluating results regarding migration history. Particularly, 1st generation migrants who recently immigrated may not be sufficiently represented which points to a general problem in public health studies [65]. Thus, associations with migration history could potentially be underestimated in our study. Moreover, the perception of delay and subsequent health risk may have been reported more or less times than actually occurred. Accordingly, a recall bias cannot be ruled out. Furthermore, we cannot distinguish between different health care providers and between severe and less severe health issues. The regression analyses regarding perceived health risk due to cost-related forgone care referred to a small sub-sample (*n* = 183). Thus, conclusions have to be drawn carefully. Finally, the ICC is quite low in some cases. However, small ICC values in logistic regression models are very common and do not necessarily indicate negligible effects [66].

## Conclusions

A high proportion of respondents stated that they had delayed and/or forgone medically necessary treatment over a period of 12 months, and that they often perceived a worsening of their health due to this unmet need. The frequency of unmet need, the perceived worsening of complaints as a result of not seeking treatment, and the associations with indicators of individual and regional social inequality as well as health insurance suggest tailored interventions. Generally, social inequalities should be reduced as it was shown that for deprived people forgone medical care tended to be higher in countries with larger income inequalities, irrespective of average economic standard [11]. From a health policy perspective, equal access to health care for those in equal need is an important principle of equity [67]. Therefore, providing conditions in which those with equal needs have equal opportunities to access health care is expected to reduce unmet need among deprived groups. To this end, multiple implications are possible due to the comprehensive nature of health care access. When focusing on non-chosen, barrier-related unmet need, reducing waiting times (e.g. by further development of appointment service centres) and private co-payments as well as ensuring health care provision in deprived areas can contribute to a decrease of unmet need and potential health risks. The study provided a deeper insight into mechanisms of unmet need and its consequences. This included a variety of individual social characteristics and regional deprivation by using multilevel analyses. However, further supply-side and demand-side determinants of accessibility have to be considered. Conceptual frameworks of health care access highlight additional dimensions apart from availability and affordability which are limited to abilities to reach and pay [7]. In terms of dimensions like approachability, acceptability, and appropriateness, abilities to perceive, to seek, and to engage also have to be taken into account when analysing access to health care on the whole. More in-depth studies of these mechanisms and a disaggregated approach to analyse unmet need are required to include all dimensions of health care access. Furthermore, future research should rely on longitudinal data to control for further individual characteristics (e.g. changes in employment status and other live events, pathogenesis) that may contribute to the association between unmet need and health care utilization, and should include clinical and administrative data [2, 3]. Even if this cross-sectional study only deals with a section of the broad spectrum of accessibility, important factors and associations in terms of unequal access to necessary health care were identified.

## Data Availability

The datasets used and/or analysed during the current study are available from the corresponding author on reasonable request.

## Abbreviations

EHIS: European Health Interview Survey
EU: European Union
OECD: The Organisation for Economic Co-operation and Development
SHI: Statutory health insurance
PHI: Private health insurance
EU-SILC: European Union Statistics on Income and Living Conditions
SHARE: Survey of Health, Ageing and Retirement in Europe
GP: General practitioner
CASMIN: Comparative Analysis of Social Mobility in Industrial Nations
GISD: German Index of Social Deprivation
OR: Odds ratio
CI: Confidence interval
MOR: Median odds ratio
ICC: Intraclass correlation coefficient
